# Reactive Thrombocytosis Leading to Recurrent Arterial Thrombosis Reversed by Management of a Prosthetic Joint Infection of the Hip

**DOI:** 10.7759/cureus.24166

**Published:** 2022-04-15

**Authors:** Matthias Papen, Stijn Ghijselings, Georges Vles

**Affiliations:** 1 Orthopedics, UZ Leuven, Leuven, BEL

**Keywords:** streptococcus oralis, prosthetic joint infection, recurrent arterial thrombosis, dair, reactive thrombocytosis

## Abstract

Prosthetic joint infections (PJIs) still pose a severe challenge for patients and the overall health care system. Infection, and PJI in particular, is a known cause of reactive thrombocytosis. Thromboembolic complications secondary to reactive thrombocytosis are infrequent and arterial thromboses are rarely described. We present the case of a 64-year-old female with reactive thrombosis and recurrent arterial thrombosis due to bilateral streptococcal PJI of the hip. Multiple episodes of acute ischemia of the right lower limb ultimately led to transfemoral amputation. Only after bilateral irrigation and debridement for infection control did the thrombocytosis resolve without any further thromboembolic complications. Early recognition of thrombocytosis, use of anti-platelet agents and early surgical treatment of the underlying infection (even when a conservative treatment may otherwise be considered) could have avoided this potentially life-threatening complication.

## Introduction

Prosthetic joint replacement surgery is effective at improving mobility and pain. Infection, although uncommon (1%-3%), causes substantial morbidity and most often requires surgical intervention. Conventional treatment options include a debridement, antibiotics, and implant retention (DAIR) procedure or one- or two-stage exchange arthroplasty. Long-term suppressive antibiotic therapy can be considered when surgery is contraindicated, if the patient is immobile and does not need a functional prosthesis or if they refuse surgery [1]. However, if this approach does not suffice to preserve a functioning arthroplasty or suppress symptoms of infection, surgical intervention may still be necessary.

Infection is one of the main causes of secondary or reactive thrombocytosis (RT). Although common for clonal thrombocytosis, thromboembolic complications are rarely seen with RT [2]. Additionally, venous thromboembolic complications vastly outnumber cases with arterial thrombosis in literature. What follows is a case discussion regarding a 64-year-old female with a late prosthetic joint infection (PJI) of both hips giving rise to an RT with concurrent and recurrent acute ischemia of the right lower limb ultimately leading to transfemoral amputation.

## Case presentation

A 64-year-old woman with known chronic bilateral Streptococcus oralis PJI of the hip was referred to the emergency department with acute ischemia of the right leg.

**Figure 1:**
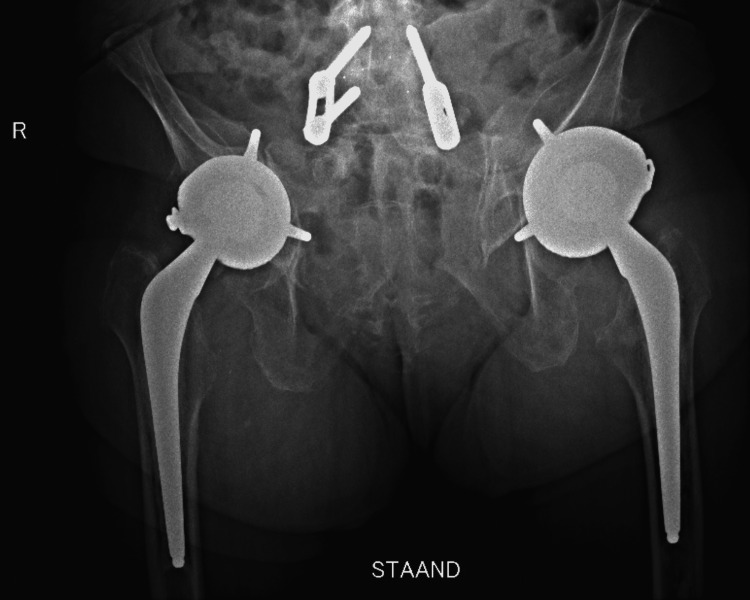
Pelvic x-ray showing bilateral total hip arthroplasty after acetabular revision surgery

Her daily medication included apixaban (Eliquis) for paroxysmal atrial fibrillation and previous coronary artery bypass grafting. She underwent a staged bilateral hip resurfacing procedure 10 years ago at a five-month interval. Both were converted to a total hip arthroplasty after two years due to symptomatic metallosis. A second revision of the right acetabular component was necessary only a few weeks later due to instability. The left acetabular component also needed revision after 6 years for similar reasons (Figure 1).

**Figure 2:**
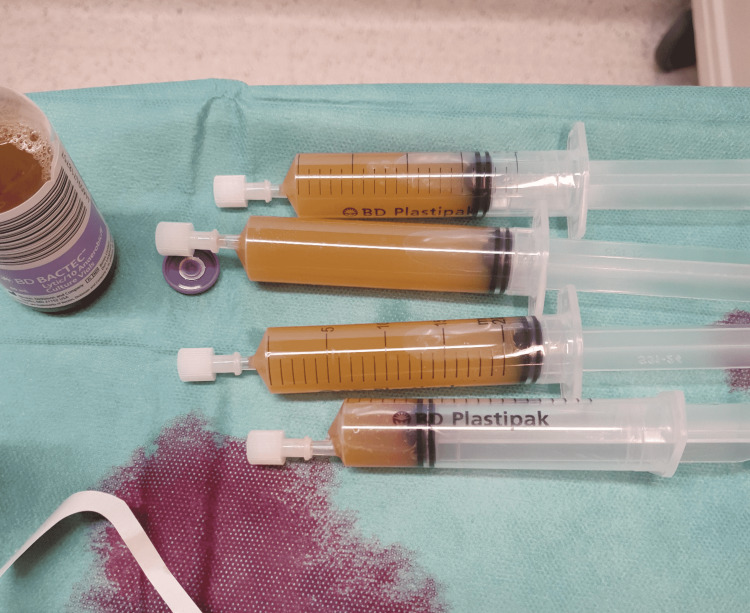
Pus-like joint aspiration fluid of both hips

Two months before referral she was admitted to the orthopedic department of a nearby hospital due to persistent hip pain for several months. C-reactive protein (CRP) measured 167 mg/L and a bilateral hip joint needle aspiration resulted in positive cultures for Streptococcus oralis (Figure 2). Two-stage revision surgery was offered but subsequently refused by the patient. Because she was allergic to penicillin, clindamycin 600mg three times a day was started as suppressive antibiotic therapy.

She then presented to the emergency department with acute ischemia of the right lower limb. Blood results showed a CRP of 118.4 mg/L and a thrombocytosis fluctuating around 700x10^9^ platelets/L. Angio-CT showed an occlusive thrombosis of the popliteal artery reaching into the anterior tibial artery and tibioperoneal trunk. She successfully underwent an embolectomy of the popliteal and anterior tibial arteries. Apixaban was switched to a therapeutic dose of low molecular weight heparins for four weeks. Despite this, she was readmitted 10 days later with a new occluding thrombus of the popliteal artery. A second thrombectomy of the popliteal artery and tibioperoneal trunk was performed, only to relapse the following day with the need for a further, third surgical intervention. Platelet inhibition in the form of aspirin 80mg once daily was added to her antithrombotic therapy.

At this point, the possibility of an underlying myeloproliferative neoplasm was investigated. Janus kinase 2 (*JAK2*) and calreticulin (*CALR*) genetic testing for essential thrombocytosis and antiphospholipid antibodies (lupus anticoagulant and anticardiolipin antibody) were negative. The parallel course of thrombocytosis and inflammation suggested a secondary thrombocytosis as part of the acute phase of infection.

A month later she was readmitted once more with acute ischemia of the right leg. Angio-CT now revealed an occlusive thrombus of the right external iliac artery with extension into the communal femoral artery and embolization of the anterior tibial and dorsalis pedis arteries. A fourth thrombectomy through the groin and dorsum of the foot seemed successful. However, due to persistent rest pain prostaglandin E1, intravenous therapy was administered and she was discharged a few days later.

**Figure 3:**
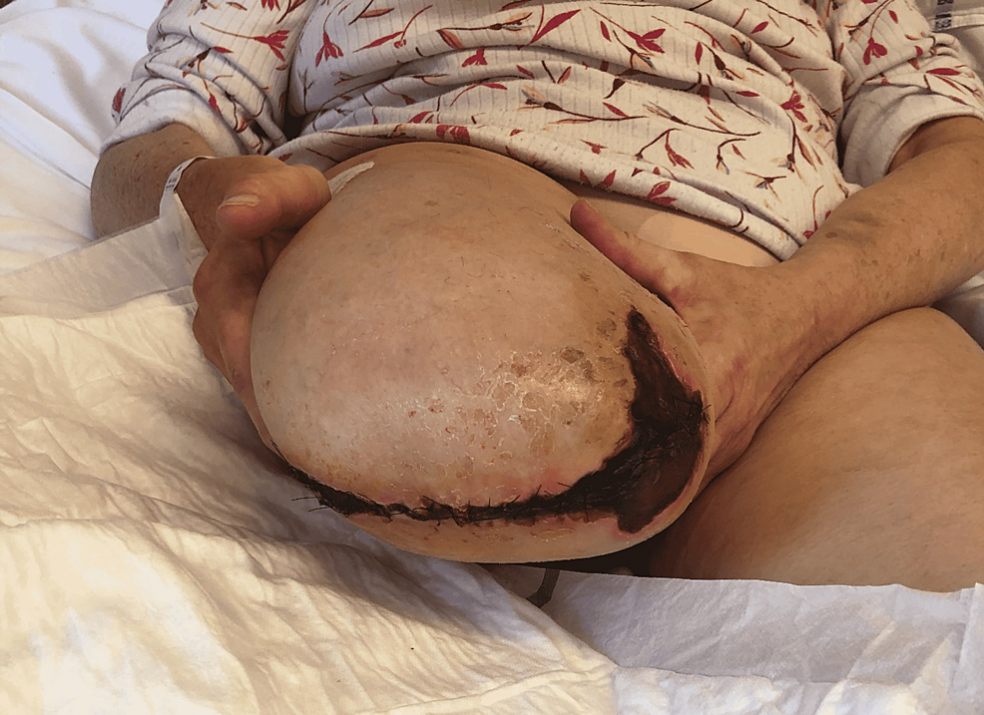
Stump four weeks after transfemoral amputation

**Figure 4:**
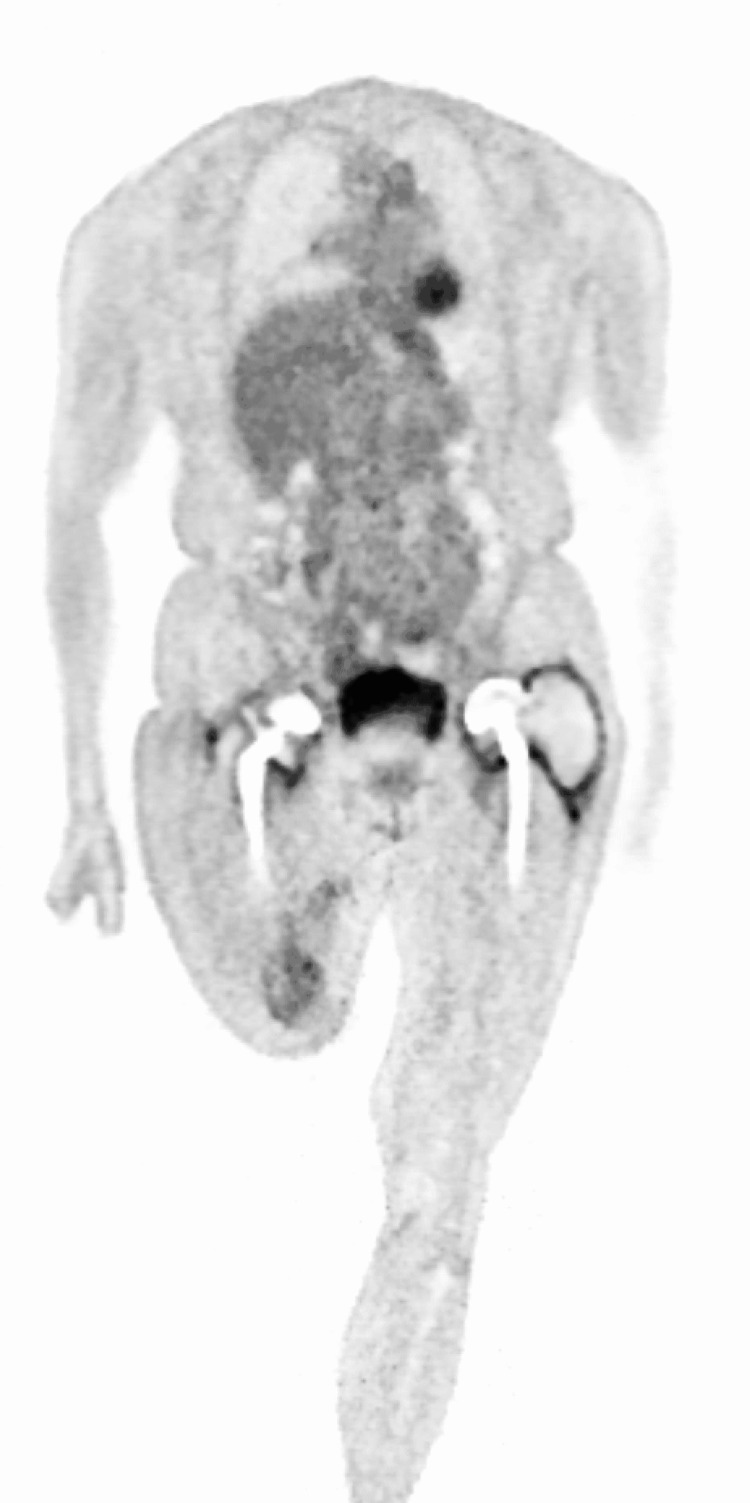
PET-CT showing pseudotumoral inflammation around the left hip and moderate metallosis around the right hip

**Figure 5:**
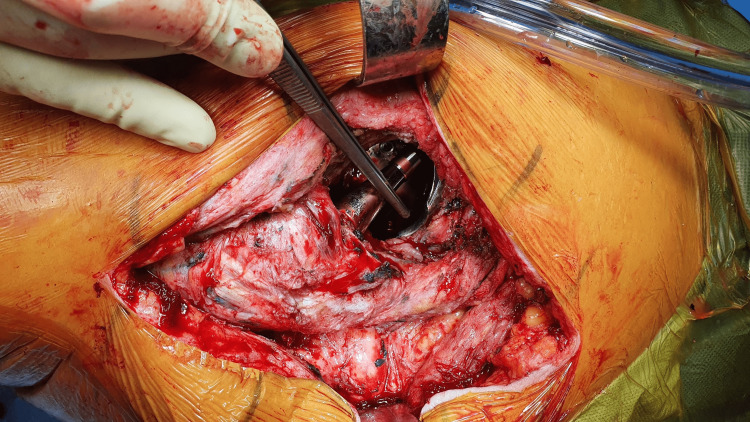
Indentation of the acetabular component due to impingement of the femoral neck after debridement of metallosis affected tissues

After only two weeks, a new episode of acute ischemia resulted in another, fifth thrombectomy of nearly all previously treated arteries. Reocclusion the following day led to the decision to perform a transfemoral amputation. The distal end of the stump developed an ischemic aspect in the first three weeks postoperatively due to a thrombus in the iliac arteries. A wait-and-see approach was maintained to await the demarcation of the ischemic zone (Figure 3). The patient was also discussed in our musculoskeletal infections multidisciplinary team meeting. No other possible explanation for the recurrent thromboembolic events had so far been found, except for an underlying reactive thrombocytosis. The patient still refused major revision surgery. However, she did agree to a DAIR procedure in an attempt to reduce or resolve chronic inflammation and subsequent thrombocytosis. Pre-operative PET-CT showed extensive periprosthetic soft-tissue inflammation matching perioperative findings of a left-sided pseudotumor and moderate metallosis around the right hip (Figure 4). Irrigation and debridement of both total hip prostheses were performed. A significant indentation at the three o’clock mark of the acetabular component (Figure 5) and a notch at the posterior side of the neck was seen. Caused by impingement, these damages are the basis of metallosis. Mobile parts were not exchanged because of unavailability. Perioperative cultures remained sterile but PCR testing was positive for the same Streptococcus species found earlier. Clindamycin suppression therapy was continued intravenously for the first two weeks postoperatively and switched back to oral suppression afterward. CT scan for evaluation of retro-acetabular pseudotumor invasion with iliac compression was negative.

CRP values continued to drop postoperatively, followed by normalization of platelet counts (PC). Wound healing of both surgical sites of the hips as well as the amputation stump was uneventful. She was able to leave the hospital six weeks after the amputation with daily wound care of the stump. Antithrombotic therapy was continued as aspirin 80mg and dabigatran (Pradaxa) 150mg, both twice daily.

The patient stopped the prescribed antibiotic course against advice three weeks prior to the follow-up appointment at two months. Nevertheless, CRP values continued to evolve favorably. A bilateral hip joint aspiration was planned to decide on further antibiotic treatment and the need for lifelong suppression. Cultures remained sterile and aspirate leukocyte counts were negative according to the 2021 EBJIS criteria for PJI [3] (right hip: 567 WBC/µL and 7.7% PMN, left hip: 702 WBC/µL and 66.2% PMN). No further antibiotic treatment was given. One more spike in her CRP values was observed, most likely related to a quickly resolving cutaneous vasculitis.

After complete healing of the stump, the patient was able to go through prosthetic rehabilitation and is now self-ambulatory using a wheelchair for outdoor displacements. After one year of post-operative follow-up, she has suffered no further thromboembolic events.

## Discussion

Chronic PJI, in this case, resulted in recurrent arterial thrombosis of the leg, leading to transfemoral amputation. The relationship between PJI and the risk of thromboembolic complications has not been well studied. Bass et al. found that the risk of venous thromboembolism within 90 days after revision of total knee arthroplasty for infection (4.3%) was twice as high when compared to aseptic revisions (2.1%) [4]. RT could be the driving force behind this elevated risk.

Infection is a known cause of RT. As such, some have suggested using a PC as an affordable adjunct diagnostic tool for PJI. Paziuk et al. concluded that when using the PC to mean platelet volume ratio in conjunction with erythrocyte sedimentation rate and CRP, diagnostic accuracy improved significantly due to its high specificity (80.85) [5].

Thromboembolic complications are a major cause of morbidity and mortality in clonal causes of thrombocytosis. Essential thrombocytosis, chronic myeloid leukemia, polycythemia vera, and primary myelofibrosis are the most common myeloproliferative neoplasms leading to thrombocytosis. With RT, on the other hand, the risk of thrombotic complications is felt to be insignificant. Other known underlying causes of RT are hyposplenism, iron deficiency, malignancy, hemolysis, tissue damage, and inflammation. It is usually considered an incidental finding with no relevant clinical implications [2]. However, severe thromboembolic complications caused by RT have been described. Outnumbered by plenty of cases of venous thrombi [6,7], only a few instances of arterial thrombosis have been published [8-10]. Up till now, the pathophysiological mechanism of platelet coagulable activity activation in RT is not well understood.

Prospective analysis of more than 100,000 people in the Copenhagen General Population Study showed a 1.8‐fold risk of cerebral arterial thrombosis associated with a high PC, excluding myeloproliferative neoplasms [11]. A prospective case-control study by Duff et al. was able to show that RT is more frequently observed in critically ill infectious patients requiring antibiotic treatment. Moreover, in vitro blood clotting tendency was associated with high PCs in a relatively linear fashion. This is of utmost importance in proving causality between RT and thromboembolism and could mean that anti-platelet agents may improve patient outcomes. Not only did patients with RT have an increased in vitro thrombotic tendency on thromboelastography, higher fibrinogen concentrations and a higher incidence of infection requiring antibiotics were seen as well when compared to patients without thrombocytosis (50% vs 27%) [12].

**Figure 6:**
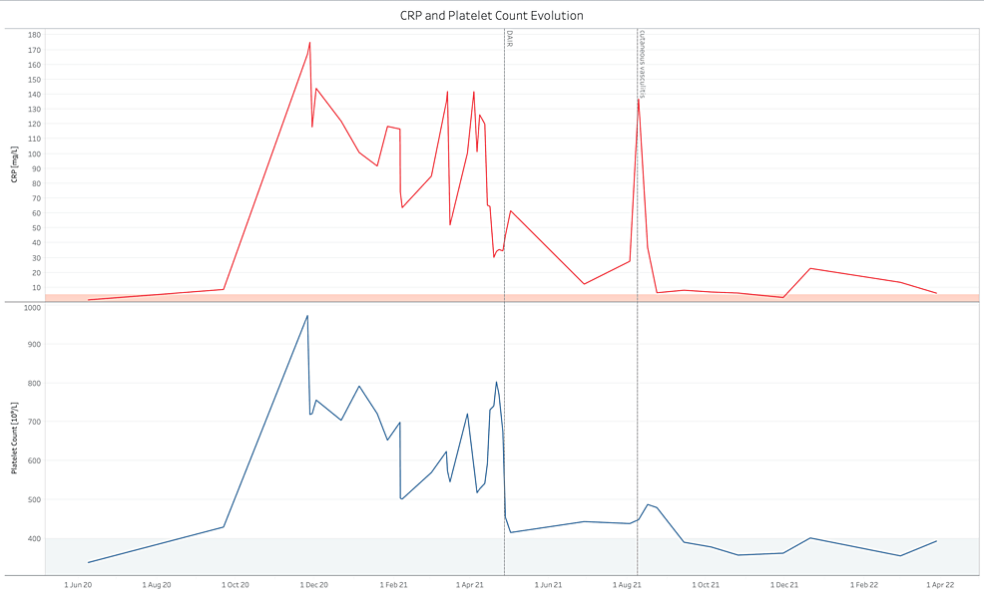
Parallel course of CRP and platelet count during follow-up of the patient

In this case, the parallel course of thrombocytosis and biochemical inflammatory parameters, a proven underlying bilateral PJI as well as negative genetic testing for a clonal cause, suggest a reactive form of thrombocytosis (Figure 6). Only in case of persistent high PCs after normalization of inflammatory markers would bone marrow examination have been indicated [2]. PJI, RT, and recurrent thromboembolic complications all resolved after a bilateral DAIR procedure, also suggesting a cause and effect relationship.

Even though many questions the efficacy of a DAIR procedure for streptococcal PJI in recent literature, infection control was successful after irrigation and debridement for this patient. Lam et al. advocate a DAIR strategy to have an acceptable success rate (83%) for early and acute hematogenous PJIs [13]. However, large cohort studies show poor results for implant retention in streptococcal PJIs, with relapse in 42.1% to 58.2% of cases [14-16], or for streptococcal PJIs regardless of surgical intervention [17]. These studies generally followed Infectious Diseases Society of America guidelines, considering DAIR treatment almost exclusively in early or acute hematogenous infections. Implant retention is not advised for chronic prosthetic infections due to maturity of the biofilm, and one- or two-stage revision is needed to eradicate the infection [1,18,19]. Irrespective of surgical treatment, long-term postoperative antimicrobial suppression has been associated with significantly better outcomes in streptococcal PJI [20].

Despite the chronicity of the symptoms in the presented case, the patient was treated with a DAIR procedure because she refused prosthesis explantation. Even in absence of suppressive antibiotics, she has no clinical or biochemical signs of reinfection one year postoperatively. This shows that even in a chronic setting, irrigation and debridement could still be of value in treating select cases of PJI where explantation or exchange arthroplasty is not an option.

## Conclusions

PJI, both acute and chronic, can lead to reactive thrombocytosis. Thromboembolic complications due to reactive thrombocytosis are relatively infrequent and not well understood in terms of the pathophysiological mechanism. Most are venous in nature, with recurrent arterial thrombosis only sporadically described. Although uncommon, this is potentially life-threatening and could be avoided by early recognition of thrombocytosis, the use of anti-platelet agents, and timely treatment of the underlying infection.

In the acute setting, a DAIR procedure is of significant value. However, recent literature has shown poor results of implant retention regarding streptococcal PJIs. Successful eradication of a bilateral chronic streptococcal PJI without long-term antibiotic suppression postoperatively in our case shows that debridement and implant retention are still a valuable option when resection or exchange arthroplasty is not possible.
